# Something Is Rotten in the State of Redox

**DOI:** 10.3390/antiox14010098

**Published:** 2025-01-16

**Authors:** Ahmad Yaman Abdin, Mohamad Jawad Nasim, Claus Jacob

**Affiliations:** Division of Bioorganic Chemistry, School of Pharmacy, Saarland University, D-66123 Saarbruecken, Germany; yaman.abdin@uni-saarland.de (A.Y.A.); jawad.nasim@uni-saarland.de (M.J.N.)

Some say that science is an art, a craft, and a passion—and, one may add these days, often also a business. In any case, it is a social process driven mostly by scientists, and thus not necessarily just an attempt to reveal the deeper secrets of nature. Science is only human, and is always open to errors, debates, and controversies. Still, there are few topics within the realm of Life Sciences which may be as controversial, and hotly debated, as the issue of antioxidants [1,2,3]. Some consider them as powerful boosters of human health, readily available from local supermarkets and greengroceries. Others are more skeptical. Indeed, mounting evidence from animal studies, human trials, and meta-analyses of the literature seems to question the beneficial impact of such antioxidants, especially orally taken ones, on animal and human health [4,5]. In some instances, these studies have even shown detrimental activity, which has led some colleagues active in the fields of redox chemistry and biology to become rather critical of the entire concept of antioxidation and redox control in health and disease.

In this Special Issue, we aim to respond to the criticisms associated with the activities of endogenous and, especially, exogenous antioxidants. The contributions address several issues at the heart of this debate, reflecting on the conceptual and semantic foundation of antioxidants, exploring the role of oxidative stress in disease pathophysiology, investigating how lifestyle factors and interventions affect the redox balance, and providing cutting-edge mechanistic and molecular insights. Together, they try to resolve some of the sticky, difficult issues surrounding the “antioxidant narrative” by looking at the use of the term itself or by considering different research programs—and outcomes—at mutually exclusive layers of scientific complexity.

In their opening Perspective, Abdin et al. discuss the potential health benefits of antioxidants from the rather unusual yet stimulating angle of language, using semantics to (re)define the various uses of the word “antioxidant” in different scientific contexts. The authors not only compare antioxidants to an English delicacy, Marmite^®^ (“you either love it or hate it”), they also use the epistemological Baumkuchen Model to demonstrate that the term “antioxidant” has been a part of the scientific language game for a few decades and is currently used differently by different scientific disciplines and at different levels of scientific complexity. Therefore, it represents not a single expression but an entire family of words with distinctively different connotations and associations. The transcendent use of this expression from a basic to a more complex discipline, such as from chemistry to physiology, is problematic as the term will receive connotations that are not empirically corroborated. This may lead to false claims and aspirations that are not warranted by the empirical data.

This Perspective sets the scene for more specific discussions on oxidative stress, antioxidants, and their role(s) in biology. Jakubek et al., for instance, in their article “Beyond Antioxidant Activity: Redox Properties of Catechins May Affect Changes in the DNA Methylation Profile—The Example of SRXN1 Gene” consider the activities associated with perceived antioxidants which may not even demonstrate any antioxidant action, looking at the different activities which may be inherent in such molecules. Similarly, Cornwell and Badiei, in their contribution “From Gasotransmitter to Immunomodulator: The Emerging Role of Hydrogen Sulfide in Macrophage Biology”, focus on the still-controversial role(s) of hydrogen sulfide (H_2_S) as an immunomodulator in macrophages. Szlachta et al., in “Serum Oxidative Status in People with Obesity: Relation to Tissue Losses, Glucose Levels, and Weight Reduction”, turn towards the layer of intact organisms, in this case, people with obesity, while other contributions consider more mechanistic aspects of oxidative stress and antioxidants in disorders, such as Cerrillos-Gutiérrez et al., in “The Inflammatory and Oxidative Status of Newly Diagnosed Class III and Class IV Lupus Nephritis” and Akhiani and Martner in “Role of Phosphoinositide 3-Kinase in Regulation of NOX-Derived Reactive Oxygen Species in Cancer”. Indeed, the possible link between oxidative stress and perceived health benefits is central to the antioxidant narrative, and research on this topic is a recurrent theme involving many different levels of scientific complexity; the research ranges from simple redox and in vitro studies to research focusing on entire human populations, obese or otherwise. One can also find animal studies touching on the oxidative stress issue, such as “Mitofilin Heterozygote Mice Display an Increase in Myocardial Injury and Inflammation after Ischemia/Reperfusion”, by Feng et al., and mechanistic intervention and interaction studies, such as “Interaction of Garcinia cambogia (Gaertn.) Desr. and Drugs as a Possible Mechanism of Liver Injury: The Case of Montelukast”, by Di Giacomo et al. Eventually, antioxidants may not be the only contributors to health, as Shen et al. show in “Reduced Ribose-5-Phosphate Isomerase A-1 Expression in Specific Neurons and Time Points Promotes Longevity in Caenorhabditis elegans”, although so far this has only been demonstrated in a worm. Indeed, together with the other contributions of this Special Issue, this manuscript promotes the longevity, not only of *C. elegans* but also of the debate on “antioxidants”, which will surely enter a second round soon!

## Data Availability

No new data were created as part of this manuscript.

## References

[1] Gladyshev V.N. (2014). The Free Radical Theory of Aging Is Dead. Long Live the Damage Theory!. Antioxid Redox Signal..

[2] Fridovich I. (1978). The Biology of Oxygen Radicals. Science.

[3] Berger R.G., Lunkenbein S., Ströhle A., Hahn A. (2012). Antioxidants in Food: Mere Myth or Magic Medicine?. Crit. Rev. Food Sci. Nutr..

[4] Ghezzi P., Ghiara V., Davies K., Sies H. (2020). Chapter 2—Epistemological Challenges of the Oxidative Stress Theory of Disease and the Problem of Biomarkers. Oxidative Stress.

[5] Ghezzi P., Jaquet V., Marcucci F., Schmidt H.H.H.W. (2017). The Oxidative Stress Theory of Disease: Levels of Evidence and Epistemological Aspects. Br. J. Pharmacol..

